# Protective Properties of *Laggera alata* Extract and its Principle Components Against d-Galactosamine-Injured Hepatocytes

**DOI:** 10.3797/scipharm.1108-16

**Published:** 2012-02-08

**Authors:** Yi-Hang Wu, Bing-Jie Hao, E Shen, Qing-Li Meng, Ming-Hui Hu, Yu Zhao

**Affiliations:** 1 Department of Pharmacy, College of Life Sciences, China Jiliang University, 310018 Hangzhou, China; 2 Intensive Care Unit, Zhejiang Provincial People’s Hospital, 310005 Hangzhou, China; 3 Department of Traditional Chinese Medicine and Natural Drug Research, Zhejiang University, 310058 Hangzhou, China

**Keywords:** *Laggera alata* extract, Isochlorogenic acid, d-Galactosamine, Hepatoprotective

## Abstract

*Laggera alata* extract (LAE) was quantitatively analyzed, and its principle components isochlorogenic acids were isolated and authenticated. Protective properties of LAE were studied using a d-galactosamine (d-GalN)-induced injury model in neonatal rat hepatocytes and a d-GalN-induced acute liver damage model in mice. Meanwhile, the effect of isochlorogenic acids derived from LAE on d-GalN-induced hepatocyte injury were also measured *in vitro*. LAE at concentrations of 10–100 μg/ml significantly reduced cellular leakage of aspartate aminotransferase (AST) and alanine aminotransferase (ALT) and improved cell viability. The isochlorogenic acids (4,5-*O*-dicaffeoylquinic acid, 3,5-*O*-dicaffeoylquinic acid and 3,4-*O*-dicaffeoylquinic acid) at concentrations of 1–100 μg/ml also remarkably improved viability of hepatocytes. The oral treatment of LAE at doses of 50, 100 and 200 mg/kg markedly reduced the serum AST and ALT activity of mice and resulted in significant recovery of hepatocytes in liver sections.

## Introduction

**Fig. 1.**
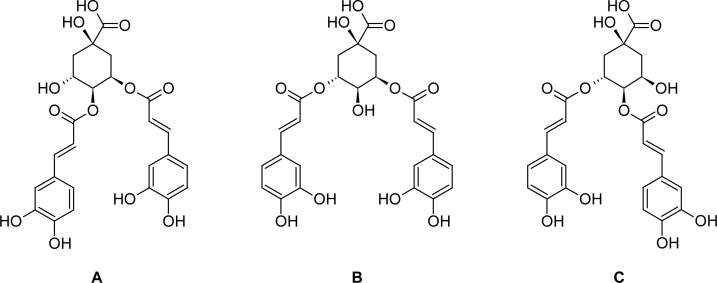
Structures of isochlorogenic acids from *L. alata*. 4,5-*O*-dicaffeoylquinic acid (**A**); 3,5-*O*-dicaffeoylquinic acid (**B**); 3,4-*O*-dicaffeoylquinic acid (**C**).

*Laggera alata* (Asteraceae) is distributed mainly in tropical Africa and Southeast Asia. This plant has been used as a folk medicine in China for over three hundred years, especially for the treatment of some ailments associated with hepatitis [1]. Most studies concerning *L. alata* focused on its folk use and phytochemical analyses [2–6]. In previous investigations, we examined the anti-inflammatory activities of *L. alata* and confirmed its potent inhibitory effects in models of acute and chronic inflammation [7]. To further validate the remarkable curative effect of *L. alata* on liver disorders, we investigated protective properties of *L. alata* extract (LAE) against d-galactosamine (d-GalN)-induced injury in primary cultured neonatal rat hepatocytes and in mice with acute hepatic injury. Also, the effect of isochlorogenic acids derived from LAE on d-GalN-induced hepatocyte injury were measured. This study is the first to find that LAE containing isochlorogenic acids (Figure 1) possesses the potent hepatoprotective effect against d-GalN-induced injury. Hence, we report the details here.

## Results and Discussion

### Effect of LAE on d-GalN-injured primary cultured rat hepatocyte

The cytotoxicity test indicated that LAE and isochlorogenic acids were almost nontoxic to the neonatal rat hepatocytes at concentrations of 1–100 μg/ml. The neonatal rat hepatocyte injury was induced by exposure to 20 mM d-GalN and the cells were subsequently treated with LAE. The results showed that LAE at concentrations of 10–100 μg/ml significantly improved cell viability and reduced cellular leakage of aspartate aminotransferase (AST) and alanine aminotransferase (ALT) (Table 1). The reduction of AST and ALT and increased cell viability provides a clear indication of the improved functional status of the cells, thus demonstrating the protective effect of LAE on chemically injured hepatocytes. Furthermore, LAE afforded much stronger protection than the reference drug silibinin.

### Effect of LAE on the mice with hepatic damage induced by d-GalN

**Fig. 2.**
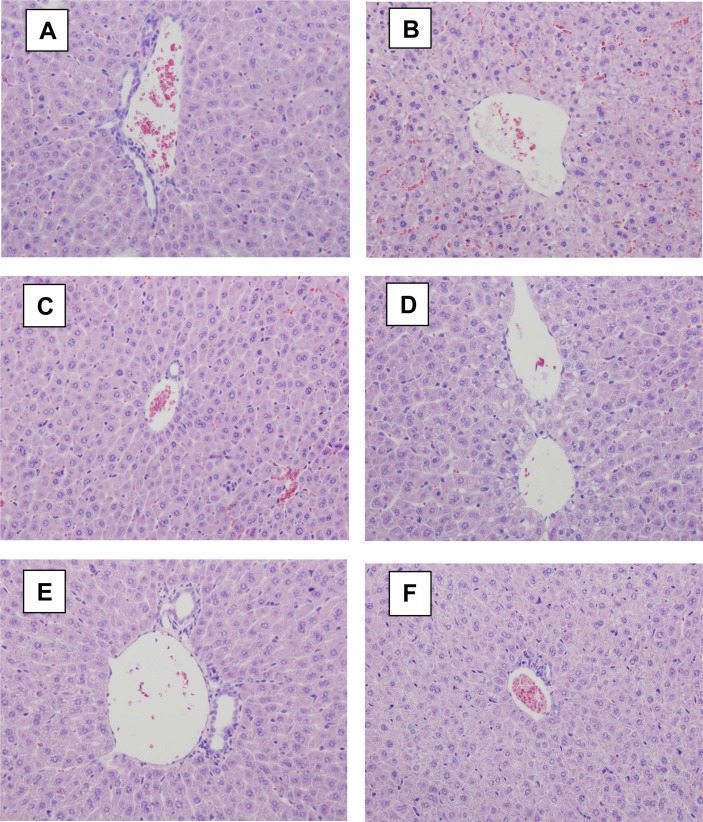
Effect of LAE on liver histopathological change of d-GalN-induced acute hepatic damage mice (HE × 40). (**A**) a control untreated mouse showing a normal central vein and hepatocytes; (**B**) a d-GalN-treated mouse showing diffuse lesions of the liver; (**C**) a silibinin (100 mg/kg)-d-GalN -treated mouse showing obvious improvement of liver damage; (**D**) a LAE (50 mg/kg)-d-GalN-treated mouse; (**E**) a LAE (100 mg/kg)-d-GalN-treated mouse, and (**F**) a LAE (200 mg/kg)-d-GalN-treated mouse. (**D**), (**E**), and (**F**) show the obvious improvement of acute liver injury.

Based on *in vitro* results, we further studied the protection afforded by LAE against d-GalN-induced acute hepatic damage in mice. The administration of d-GalN caused diffuse lesions of the liver (multiple patchy hepatocyte necrosis in the centrilobular zone and little hepatocyte steatosis etc.), which appeared in all animals of the model control group (Figure 2). It is suggested that the success ratio of acute liver damage model induced by d-GalN was 100% in the model group. No histological abnormalities were observed in vehicle control mice. The oral treatment of LAE at doses of 50, 100 and 200 mg/kg significantly reduced the serum AST and ALT levels of the mice with hepatic damage (Table 2). Also, the administration of different doses of LAE resulted in significant recovery of hepatocytes in different sections of the liver (Figure 2). LAE at doses of 100 and 200 mg/kg showed near normalization of the tissues. The ten mice of the LAE (50 mg/kg)-treated group also showed obvious improvement of liver damage. Moreover, LAE afforded much stronger protection than the reference drug silibinin. The results suggested LAE markedly ameliorated d-GalN-induced acute hepatic damage, consistent with the results of *in vitro* research.

### Effect of isochlorogenic acids on d-GalN-injured primary cultured rat hepatocytes

To further demonstrate the substance basis responsible for the bioactivity of LAE, hepatoprotection of its principle components isochlorogenic acids were studied using d-GalN-induced injury model in primary cultured neonatal rat hepatocytes. The results showed that isochlorogenic acids (3,4-*O*-dicaffeoylquinic acid, 3,5-*O*-dicaffeoylquinic acid and 4,5-*O*-dicaffeoylquinic acid) significantly improved cell viability at concentrations of 1–100 μg/ml (Table 3). Furthermore, isochlorogenic acids afforded much stronger protection than the reference drug silibinin at a concentration of 100 μg/ml, thus suggesting that isochlorogenic acids play an important role in the bioactivity of LAE.

Hepatitis implies injury to the liver characterized by the presence of inflammatory cells in the tissue of the organ. So far, there is no suitable drug to treat patients with hepatitis. d-GalN is often used in pharmacodynamics research to induce hepatic injury because this model of liver damage most closely resembles the changes observed during human hepatitis [8, 9]. Free radicals are toxic to hepatocytes and initiate a reactive oxygen species-mediated cascade causing hepatocyte cell death and leading to acute hepatitis [10, 11]. Oxygen-derived free radicals released from activated hepatic macrophages are the primary cause of d-GalN-induced liver damage [12, 13]. Also, increased production of reactive oxygen species has been reported in primary culture of rat hepatocytes damage induced by d-GalN [14]. In some pathological conditions, natural products can play an important role in two aspects: enhance the activity of original natural antioxidants and neutralize reactive oxygen species by nonenzymatic mechanisms [15]. d-GalN-induced hepatocyte injury is closely related to the formation of oxidative stress. LAE improved d-GalN-induced hepatocyte injury, thus suggesting its ability to ameliorate oxidative stress. Hence, hepatoprotection of LAE may be achieved by scavenging free radicals to ameliorate oxidate stess from d-GalN-induced injury in hepatocytes.

The analysis results of LAE showed that this extract has a high content of phenolic compounds, especially isochlorogenic acids such as 3,4-*O*-dicaffeoylquinic acid, 3,5-*O*-dicaffeoylquinic acid and 4,5-*O*-dicaffeoylquinic acid. Isochlorogenic acids exhibit several pharmacological activities such as antioxidative, anti-inflammatory and antiviral effects etc. [16–18]. In this study, hepatoprotective effects of isochlorogenic acids from *L. alata* have been confirmed and more detailed studies on their action mechanism are currently in progress. In addition, the significant concentration ranges of both LAE (10–100 μg/ml) and isochlorogenic acids (1–100 μg/ml) are similar, but the content of isochlorogenic acids in LAE is limited. It is probably associated with the synergetic actions of different isochlorogenic acids and/or the effects of the other coexisting components of LAE. Hepatoprotectors are necessary in the treatment of human hepatitis. The beneficial role of hepatoprotectors in viral hepatitis is achieved by its inhibitory action on inflammatory and cytotoxic cascade of events induced by the viral infection. Also, it can improve the regeneration process and normalize the liver enzymes by its action on protein synthesis [19]. Taken together, LAE may be a potent hepatoprotector and isochlorogenic acids may be its major active compounds responsible for the biological activity.

In conclusion, the study firstly verifies the potent hepatoprotective effect of *L. alata* extract containing isochlorogenic acids against d-GalN-induced hepatocyte injury *in vitro* and *in vivo.* Isochlorogenic acids may be its substance basis responsible for the hepatoprotective potential. Hepatoprotective effect of LAE is probably associated with scavenging free radicals to ameliorate oxidate stess. These data provide a scientific explanation for the folk uses of *L. alata* in the treatment of some ailments associated with inflammation including hepatitis.

## Experimental

### Chemicals

AST and ALT diagnostic kits, 3-(4,5-dimethylthiazol-2-yl)-2,5-diphenyltetrazolium bromide (MTT), d-GalN and silibinin were purchased from Sigma Chemical Co. (St Louis, MO, USA). Fetal bovine serum, 1640 medium and tris base were purchased from Gibco-BRL (Grand Island, NY, USA). All other reagents were of the highest commercial grade available.

### Experimental animals

Male ICR mice weighing 20–25 g and 3-day-old Sprague-Dawley rats were obtained from the Experimental Animal Center of Zhejiang Province. ICR mice were kept in a room maintained at 22 ± 2°C and at relative humidity between 40% and 70%.

### Preparation of plant extract and isochlorogenic acids

*Laggera alata* (DC.) Sch.Bip. ex Oliv. was collected from Tengchong county, Yunnan Province, China. Plant sample was authentified and a voucher specimen was deposited in the herbarium of College of Pharmaceutical Sciences, Zhejiang University. *L. alata* extract (LAE) was prepared and its principle components were quantitatively analyzed according to the method we reported previously [7]. In brief, the dried material (10 kg) was extracted with 95% ethanol. The extract was concentrated and then dissolved in hot water. This solution was basified up to pH 9–10 with 5% sodium carbonate, followed by repeated extraction with ethyl acetate. The aqueous extract left was further partitioned with *n*-butanol after acidification to pH 4 using 1N hydrochloric acid. The *n*-butanol fraction was washed with water to pH 7 and condensed to afford a dark-brown powder (150 g), which was named LAE. Quantitative analysis of LAE led to a conclusion that this extract fraction has a high content of phenolic compounds that made up half of the extract [52.6 g GAE(gallic acid equivalents)/100 g extract]. The HPLC analyses indicated that isochlorogenic acids (4,5-*O*-dicaffeoylquinic acid, 3,5-*O*-dicaffeoylquinic acid and 3,4-*O*-dicaffeoylquinic acid) were the major components in LAE whose content amounted to 51%. Isochlorogenic acids were isolated and authenticated as described in the method reported [20, 21]. LAE was dissolved in 0.5% CMC-Na solution to be administered to the tested animals.

### Primary culture of neonatal rat hepatocytes

Hepatocytes were isolated from 3-day-old Sprague-Dawley rats according to the method of Anil Kumar [22]. Cell viability, determined by trypan blue exclusion assay, was over 90%. The isolated hepatocytes were suspended in 1640-medium, and then transferred to 96-well culture plates at a density of approximately 5 × 10^5^ cells/ml. After plating, the cells were incubated at 37°C (95% humidity, 5% CO_2_). After hepatocyte attachment to the culture plate, the medium was exchanged to remove unattached or dead cells. Twelve hours later, the hepatocytes were treated with hepatotoxic agents and tested in the following assays.

### Cytotoxicity of LAE and isochlorogenic acids

Cytotoxicity induced by LAE or isochlorogenic acids treatment was measured using the MTT assay as follows: Hepatocytes were cultured in 1640-medium in the presence of 1–100 μg/ml LAE for 48 h and then 10 μl of MTT (5 mg/ml) was added to cells in each well. After 4 h of culture, the medium was removed, and the blue formazan crystals that had formed were dissolved in dimethyl sulfoxide. The absorbency of formazan generated from MTT was measured at 570 nm using a Synergy™ HT Multi-Mode Microplate Reader. Cell survival was defined as the amount of formazan production relative to that by cells not treated with hepatotoxic chemicals, and expressed as a percent.

### Effect of LAE and isochlorogenic acids on d-GalN-induced hepatocyte damage

After the hepatocytes had been incubated for 8 h with 20 mM d-GalN, the cells were then incubated for another 48 h in fresh culture medium containing 1–100 μg/ml LAE. Silibinin at a concentration of 100 μg/ml was used as a reference drug. Hepatocyte injury was assessed by measuring the amount of AST and ALT leakage as well as cell viability. AST and ALT leakages into the medium were quantified using diagnostic kits for each enzyme. Viability was calculated as described above. In addition, the effect of isochlorogenic acids (4,5-*O*-dicaffeoylquinic acid, 3,5-*O*-dicaffeoylquinic acid and 3,4-*O*-dicaffeoylquinic acid) on d-GalN-induced hepatocyte injury were also measured at concentrations of 1–100 μg/ml as above. The hepatoprotection of isochlorogenic acids was evaluated by cell survival (the absorbency of formazan generated from MTT at 570 nm).

### Effect of LAE on d-GalN-induced acute hepatic damage in mice

Animals were divided into 6 groups comprising ten mice in each group. Normal and model control groups received 0.5% CMC-Na solution at a dose of 10 ml/kg. Drug control group received silibinin at a dose of 100 mg/kg. Experimental drug groups received LAE at doses of 50, 100, and 200 mg/kg, respectively. The vehicle and drugs were administered orally to the groups of mice, respectively, once per day for 7 days. One hour after the last administration, liver damage was induced in mice of model and drug groups by intraperitoneal injection of d-GalN at a dose of 850 mg/kg. Twenty-four hours after the last administration, mice were slightly anaesthetized with ether and blood samples were taken from the eyepit. The serum was separated for the measurement of AST and ALT. The serum AST and ALT activity were determined using the AST and ALT detection kits, respectively. For histopathological analysis, liver specimens were stained with hematoxylin and eosin (HE).

### Data analysis

Experimental data were expressed as mean ± standard deviations and subjected to a one-way analysis of variance (ANOVA) and Student’s t-test. P<0.05 was chosen as the criterion of statistical significance.

## Figures and Tables

**Tab. 1.** Effect of LAE on d-GalN-injured primary cultured rat hepatocyte.

**Group**	**Concent. (μg/ml)**	**Absorbency (570 nm)**	**Cell survival (%of vehicle)**	**AST (IU/L)**	**ALT (IU/L)**
Vehicle	–	0.834 ± 0.042**	100	49.66 ± 2.08**	32.85 ± 2.56**
d-GalN-Control	–	0.158 ± 0.008	18.94	98.33 ± 5.01	59.36 ± 3.79
Silibinin	100	0.367 ± 0.027**	44.00	70.33 ± 3.05*	41.28 ± 4.78
LAE	1	0.175 ± 0.025	20.98	86.22 ± 7.25	53.39 ± 5.18
	10	0.375 ± 0.016**	44.96	69.44 ± 4.78*	42.11 ± 2.69*
	50	0.542 ± 0.026**	64.99	60.22 ± 4.01**	40.28 ± 4.12*
	100	0.676 ± 0.025**	81.06	52.15 ± 2.87**	36.21 ± 3.03**

Values are expressed as mean ± SD of six replicates.

* *P*<0.05 and

** *P*<0.01 represent the significance of the difference from the d-GalN control. Silibinin is used as positive control.

**Tab. 2.** Effect of LAE on the serum AST and ALT activity of mice with hepatic damage induced by d-GalN.

**Group**	**Dose (mg/kg)**	**AST (IU/l)**	**ALT (IU/l)**
Vehicle	–	48.65 ± 6.78**	28.59 ± 3.15**
Model	–	124.37 ± 10.35	75.16 ± 5.79
Silibinin	100	85.94 ± 5.63*	50.19 ± 4.29*
LAE	50	65.38 ± 4.59**	45.31 ± 6.12*
	100	60.79 ± 6.96**	38.33 ± 5.11**
	200	56.88 ± 5.61**	36.99 ± 4.77**

Values are expressed as mean ± SD of ten mice.

* *P*<0.05 and

** *P*<0.01 represent the significance of the difference from the model control. Silibinin is used as positive control.

**Tab. 3.** Effect of isochlorogenic acids on survival of d-GalN-injured neonatal rat hepatocyte.

**Group**	**Concentration (μg/ml)**	**Absorbency (570 nm)**	**Cell survival (% of vehicle)**
Vehicle	–	0.931 ± 0.058**	100
d-GalN-Control	–	0.304 ± 0.041	32.65
Silibinin	100	0.493 ± 0.039**	52.95
3,4-dicaffeoylquinic acid	1	0.460 ± 0.074**	49.41
	10	0.470 ± 0.067**	50.48
	50	0.492 ± 0.064**	52.85
	100	0.613 ± 0.011**	65.84
3,5-dicaffeoylquinic acid	1	0.474 ± 0.061**	50.91
	10	0.476 ± 0.022**	51.13
	50	0.563 ± 0.0683**	60.47
	100	0.643 ± 0.017**	69.06
4,5-dicaffeoylquinic acid	1	0.415 ± 0.047*	44.58
	10	0.491 ± 0.054**	52.74
	50	0.537 ± 0.007**	57.68
	100	0.597 ± 0.012**	64.12

Values are expressed as mean ± SD of six replicates.

* *P*<0.05 and

** *P*<0.01 represent the significance of the difference from the d-GalN control. Silibinin is used as positive control.
